# Prediction of Tool Forces in Manual Grinding Using Consumer-Grade Sensors and Machine Learning

**DOI:** 10.3390/s21217147

**Published:** 2021-10-28

**Authors:** Matthias Dörr, Lorenz Ott, Sven Matthiesen, Thomas Gwosch

**Affiliations:** Karlsruhe Institute of Technology (KIT), 76131 Karlsruhe, Germany; lorenz.ott@web.de (L.O.); sven.matthiesen@kit.edu (S.M.); thomas.gwosch@kit.edu (T.G.)

**Keywords:** inertial measurement unit, force estimation, data logger, tool forces, manual grinding, Gaussian process regression, artificial intelligence, hand-held power tool, angle grinder, machine learning regression

## Abstract

Tool forces are a decisive parameter for manual grinding with hand-held power tools, which can be used to determine the productivity, quality of the work result, vibration exposition, and tool lifetime. One approach to tool force determination is the prediction of tool forces via measured operating parameters of a hand-held power tool. The problem is that the accuracy of tool force prediction with consumer-grade sensors remains unclear in manual grinding. Therefore, the accuracy of tool force prediction using Gaussian process regression is examined in a study for two hand-held angle grinders in four different applications in three directions using measurement data from an inertial measurement unit, a current sensor, and a voltage sensor. The prediction of the grinding normal force (rMAE = 11.44% and r = 0.84) and the grinding tangential force (rMAE = 18.21% and r = 0.82) for three tested applications, as well as the radial force for the application *cutting with a cut-off wheel* (rMAE = 19.67% and r = 0.80) is shown to be feasible. The prediction of the guiding force (rMAE = 87.02% and r = 0.37) for three tested applications is only possible to a limited extent. This study supports data acquisition and evaluation of hand-held power tools using consumer-grade sensors, such as an inertial measurement unit, in real-world applications, resulting in new potentials for product use and product development.

## 1. Introduction

Manual grinding with hand-held grinders is performed in metalworking and construction. For the manual grinding process, the forces between the tool and the workpiece, known as the tool forces, are of great importance. The knowledge of these forces can be used, for example, to determine the productivity by means of material removal and machined surface quality [1], as well as the quality of the work result [2]. Knowledge of tool forces enables many improvements in product development and the use of hand-held grinding machines. In the area of reliability engineering (Design for Reliability), a good knowledge of the external forces and, thus, of the tool forces acting on the product is essential [3]. In addition, the knowledge of the forces in real time enables further product or process improvements such as the adjustment of the control or predictive maintenance algorithms based on the predicted tool force data. 

The problem is that the tool forces of hand-held grinding machines cannot be measured directly in the grinding contact [4]. The determination of tool forces, for example by modeling or sensor integration, is a challenge for hand-held power tools, especially when the motion is not clearly defined [5]. 

In the automated grinding process, the tool or workpiece is directed and controlled by a machine or a robot. In this area, the tool forces are determined either by measuring directly with rigidly connected force sensors on the tool or the workpiece or indirectly by modeling the work process and predicting the tool force. For prediction of the tool force, empirical models, numerical models, analytical models, and artificial intelligence models including machine learning models are used [6,7]. Overall, machine learning models show promising results in predicting tool forces [8]. Both the setpoint variables and measured variables from sensors are used as input variables for the models. For the prediction, sensors are often additionally integrated to measure the displacement of the working shaft [9] or to measure the acoustic emission [10]. An extension to predicting tool forces in automated grinding is the use of virtual sensors, whose measured values are predicted from physical quantities [11].

The manual grinding process with hand-held power tools is highly affected by the user [12]. Therefore, the process of manual grinding, as opposed to automated grinding, depends on the knowledge, skills, posture, gripping forces, and personal strength of the user [13]. This means that the user influences the grinding tangential force and grinding normal force, hence the consistency of the whole grinding process [14]. In addition, the grinding forces are influenced by grinding disc wear and manual feed [1]. The correlation between the average tool forces with grinding process parameters indicates that manual grinding with hand-held power tools is a force-controlled process and not a path-controlled process like automated grinding [15]. Therefore, the approaches to determining tool forces in the field of automated grinding are not transferable to hand-held machines without further investigation.

In the field of manual grinding, measurement of the tool forces is mainly done on stationary test setups where a force sensor is rigidly placed between the workpiece and the test setup [16,17,18]. However, the use of stationary test setups limits the force measurement to laboratory tests. A transfer to field tests with arbitrary workpieces is not possible. An approach is to measure the acceleration at the handle of a hand-held grinding machine or workpiece to be machined and to calculate the tool forces using an experimentally determined transfer function [4]. With this approach, only high-pass filtered tool forces and no stationary ones can be determined. A different approach is to calculate the forces from the displacement of the working shaft, which can be determined with non-contact sensors via the stiffness of the rolling bearings [19,20]. A similar approach to determine the forces on the drive train is to integrate force sensors into the housing of an angle grinder [5]. However, with both approaches, only the forces on the drive train and not the tool forces at the tool-workpiece contact can be measured, since the transmission behavior of the grinding disc tool must be considered [21]. Besides, the integration of force sensors requires structural changes of the hand-held grinding machine, which affects the work process of the user. In addition, a verification of the force measurement for angle grinders at operating points over 2000 rpm and over 7 N has not been investigated yet [22].

In other fields, determining forces with machine learning methods based on measurement data from consumer-grade sensors, e.g., from an inertial measurement unit (IMU), is a common problem. Thus, body forces and motions, such as joint forces [23,24], joint torques [25], locomotor intentions [26], center of mass-center of pressure inclination angle [27], and the ground reaction force [28] have been predicted using one or more IMUs. The results indicate that the prediction of the motion and forces of a user is in principle also possible in manual grinding with an IMU. However, the prediction of tool forces is strongly disturbed by the interaction with the hand-held grinding machine and the machining process. Therefore, it is hypothesized that in addition to the data from an IMU, the rotational speed and electrical power of a hand-held grinder must be used as relevant input parameters for tool force prediction.

The prediction of tool forces for hand-held grinding machines with machine learning has been illustrated in one study and a preliminary study by the authors. In the study [29], tool forces on a hand-held grinding machine (Dremel) are predicted via a binary classification into a high force range (4–8 N) and a low force range (0–2.5 N) using a support vector machine (SVM) with an accuracy of 79.58%. The input data for the classification was measured with an accelerometer (three directions), a gyroscope (three directions), an acoustic emission sensor (20–500 kHz), and a current sensor. In a preliminary study by the authors [18], the prediction of tool forces for hand-held grinding machines (corded angle grinder with universal motor) was performed through regression with decision forests using the bagging algorithm. The algorithm was applied to four applications (different grinding discs), each consisting of four trials. An average accuracy was obtained, reported with a coefficient of determination (R^2^) of R^2^ = 0.33 for the guiding or axial force, of R^2^ = 0.91 for the tangential grinding force and of R^2^ = 0.97 for the normal grinding force. The rotational speed of the tool, the electrical power, and the acceleration at the gearbox housing of the angle grinder were measured with laboratory measurement equipment that had a sampling frequency of 20 kHz as input for the regression. 

In both studies, the validation was performed through cross-validation (CV), and not via leave-one-out cross-validation. Therefore, the transferability of the accuracy to similar unknown trials remains uncertain. For real-world use, it is necessary to use consumer-grade sensors instead of wired laboratory measurement technology. The problem is that uncertainty remains in how accurate the prediction of tool forces will be with consumer-grade sensors, such as an IMU or a shunt, at lower sampling frequencies in leave-one-out cross-validation.

Therefore, this study aims to investigate the accuracy of tool force prediction during manual grinding by using measurement data from consumer-grade sensors at lower sampling frequencies. For this purpose, a data logger that includes an IMU, a current sensor, and a voltage sensor operating at a sampling frequency of 1000 Hz as well as a machine learning model developed and optimized in this work are used. The accuracy of tool force prediction in three directions using the developed machine learning model is investigated for two hand-held grinding machines, four different applications, and three different sampling frequencies of predicted tool forces. Predicting tool forces with a machine learning model using consumer-grade sensors enables data acquisition in real-world applications, which results in new potentials for the manual grinding process.

## 2. Materials and Methods

### 2.1. Experimental Study

**Figure 1:**
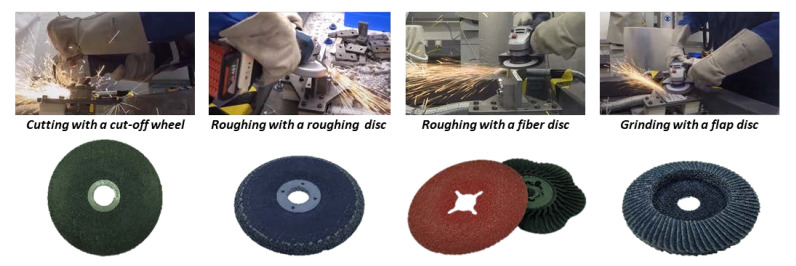
Four different grinding discs and applications considered in the study.

The objective of the study is to measure the tool forces as dependent variables and the operating parameters of an angle grinder as independent variables for the machine learning model. A total of 64 tests were carried out on two different cordless angle grinders CCG18 (CCG18-125 BL, C. & E. Fein GmbH, Schwäbisch Gmünd, Germany) and GWS18 (GWS18-125 V-LI, Robert Bosch GmbH, Leinfelden-Echterdingen, Germany) by one test person. A setpoint pressure force was given to the test person and displayed on a screen with limits of +/− 2 N during the trial. Despite the setpoint pressure force, the pressure force has a high dynamic component that exceeds the set point. After four trials, a longer break was taken to give the test person time to recover. During the tests, a total of four different applications were tested: *Cutting with a cut-off wheel*, *roughing with a roughing disc*, *roughing with a fiber disc,* and *grinding with a flap disc*. Figure 1 shows the four different applications and the used grindings discs.

The application *cutting with a cut-off wheel* was performed using a corundum cut-off wheel to cut a flat steel sample with dimensions of 80 mm × 80 mm × 10 mm until the sample was completely cut in half. The cut-off wheels were changed after two trials. The application *roughing with a roughing disc* was performed with a corundum roughing disc. The application *roughing with a fiber disc* was performed with a ceramic fiber disc. For both applications, a narrow steel sample with dimensions of 200 mm × 10 mm × 80 mm was machined along the edges to simulate the machining of a weld seam (roughing). For both applications, the discs were changed after four trials. The application *grinding with a flap disc* was performed with a flap disc on a flat steel sample measuring 120 mm × 80 mm, using circular movements. The flap discs were changed after four trials. The setpoint contact for all trials was 25 N. Before the tests, the discs were run in for 120 s.

### 2.2. Test Setup for Force Measurement and Data Loggers

For the measurement of tool forces, a test setup was used, which allows the steel samples to be fixed in order to carry out the four applications presented. Three three-dimension piezo force sensors (9347C, Kistler Instrumente GmbH, Sindelfingen, Germany) were used to measure the dynamic forces on a steel sample in three dimensions. The test setup and the steel samples allow the measurement of tool forces up to 500 Hz without natural frequencies. The forces were measured with a sampling frequency of 20 kHz.

**Figure 2:**
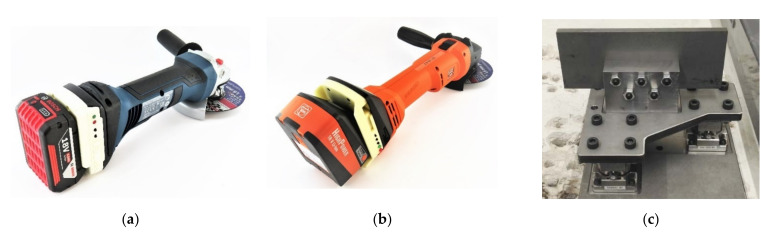
Used angle grinders with matching data logger and test setup for measuring the tool forces: (**a**) Angle grinder GWS18(**b**), angle grinder CCG18, and (**c**) test setup with the steel sample for the applications *roughing with a roughing disc* and *roughing with a fiber disc*.

For measurement of the operating parameters of both angle grinders, a data logger, which was mounted at the intermediate point between battery and angle grinder, was used. The concept and a comparable version of the data logger were published in [18,30]. The data logger consists of a 3D printed housing, a microcontroller (Cortex-M0, ARM Limited, Cambridge, United Kingdom) and several sensors: An IMU (LSM9DS0, STMicroelectronics, Geneva, Switzerland) was used to measure acceleration, angular velocity, and the magnetic field in all three directions. A current shunt monitor (INA 169, Texas Instruments, Dallas, TX, USA) was used to measure current on the angle grinder GWS18, a Hall effect sensor (ACS758-100U, Allegro MicroSystems, Manchester, New Hampshire, United States) was used to measure current on the angle grinder CCG18, and a voltage divider was used for voltage measurement. The time series were measured at a frequency of 1000 Hz, time-stamped using a real time clock, and stored on an SD card. The two angle grinders, their matching data logger, and the test setup with the steel sample for the applications *roughing with a roughing disc* and *roughing with a fiber disc* are shown in Figure 2.

### 2.3. Data Processing

**Figure 3:**
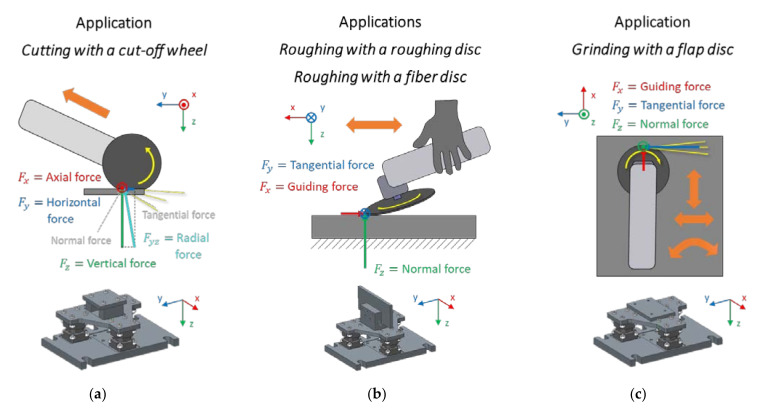
Transformation of the forces measured at the test setup to the tool forces for all applications: Transformation (**a**) for application *cutting with a cut-off wheel*, (**b**) for applications *roughing with a roughing disc* and *roughing with a fiber disc* and (**c**) for application *grinding with a flap disc*.

For the tool forces, nine time series were measured with the test setup at a sampling frequency of 20 kHz. The data processing of the tool forces was conducted by adding the time series of the three force sensors from the same directions (X-Axis, Y-Axis, Z-Axis) to compensate for constraint forces. The transformation of the forces measured at the test setup to the tool forces is shown in Figure 3. 

For *cutting with a cut-off wheel*, the force in the X-direction is the axial force $F_{axial,cut}$. The radial force $F_{radial,cut}$ for *cutting with a cut-off wheel* combines the tangential force and the normal force, as the direction changes due to the changing angle between angle grinder and workpiece. The radial force $F_{radial,cut}$ is formed by the addition of the measured vertical and horizontal force. Moreover, for *roughing with a fiber disc*, the force in the X-direction is the guiding force $F_{guiding,fiber}$ [31]. For *roughing with a fiber disc*, the force in the Y-direction is the grinding tangential force $F_{tangential,fiber}$, and the force in the Z-direction is the grinding normal force $F_{normal,fiber}$. For *roughing with a roughing disc*, the force in the X-direction is also the guiding force $F_{guiding,roughing}$, the force in the Y-direction is the grinding tangential force $F_{tangential,roughing}$, and the force in the Z-direction is the grinding normal force $F_{normal,roughing}$. Lastly, for *grinding with a flap disc*, the stationary forces X- and Y-direction are more volatile due to the circular swinging movement of the angle grinder and the stationary coordinate system of the test setup. Nevertheless, the force in X-direction is the guiding force $F_{guiding,flap}$, the force in the Y-direction is the grinding tangential force $F_{tangential,flap}$, and the force in the Z-direction is the grinding normal force $F_{normal,flap}$. One trial of roughing with a fiber disc was not evaluated for the GWS18 because the long machining time combined with the high load resulted in repeated power interruptions to the motor during this trial.

**Figure 4:**
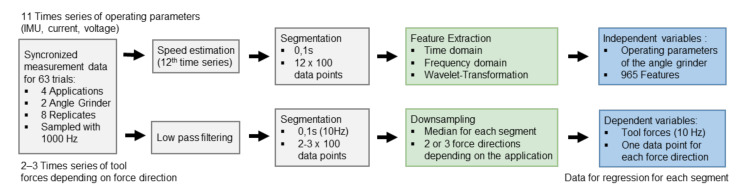
Data processing steps for independent variables (operating parameters measured by data logger) and the dependent variables (tool forces measured by test setups).

To match the sampling rate to the data logger data, the time series of the tool forces were downsampled to 1000 Hz. Then, the time series of the tool forces were synchronized with the time series of the operating parameters measured by the data logger. All phases outside of one second before and after tool-workpiece contact were removed. Figure 4 shows the data processing steps to extract the independent and dependent variables for regression. 

No filters were used in the data processing of the time series of the operating parameter measured by the data logger. From the triaxial measurement signals of the acceleration, a ‘virtual’ speed signal of the working shaft was calculated via a Fast Fourier Transform (FFT), since the speed is a relevant variable for the operating point of an angle grinder. The time series of signals for predicting the tool forces are acceleration (three directions), angular velocity (three directions), magnetic field (three directions), current, voltage, and speed. We used constant linear segmentation to split the time series into a subset of smaller time series of 0.1 s (segments). On the obtained segments, the features were calculated independently. Therefore, each time series was mapped into a feature space. Overall, 965 features were extracted. Among these features were the following:

(a) Minimum, maximum, sum, abs sum, mean, median,
(b) standard deviation, root mean square, interquartile range, skewness, several percentiles (5%, 20%, 80%, 95%),
(c) peak2peak, peak2rms, margin factor, root sum of squares, absolute root mean,
(d) zero-crossing rate, mean crossing rate,
(e) mean frequency, median frequency,
(f) value and frequency of the three highest peaks in amplitude spectrum,
(g) spectral energy in 20 defined sections of the amplitude spectrum (e.g., 80–150 Hz, 350–500 Hz) and
(h) several Daubechies wavelets (e.g., db2_Wavelets_approx, db2_Wavelets_shannon_Entropy3).

After extraction, all features were standardized (mean value equals 0 and standard variation equals 1) and post-processed. The post-processing steps include a check for missing values and zero values among each feature. In the case of more than 5% missing values, features are removed. This is also done for features that include more than 30% zeros. Spline interpolation was used to estimate values for features with less than 5% missing values. 

In the data processing of the tool forces, a low-pass filter of 100 Hz (Butterworth, 4th order) was used. The time series were then split into a subset of segments using constant linear segmentation. For each segment, the medians of the tool forces were calculated, serving as outcome variables (dependent variable) for prediction. Three segment lengths of 0.1 s (100 data points per signal and segment), 1 s (1000 data points per signal and segment), and 0.05 s (50 data points per signal and segment) were varied in this study to determine the accuracy of the predicted tool forces at different sampling frequencies. Thus, the prediction of tool forces is carried out with sampling frequencies of 10 Hz, 1 Hz, and 20 Hz. 

### 2.4. Regression 

In statistical modeling, regression analysis is used to estimate the relationship between a dependent variable Y $={\left[y_{1\;},\;y_{2\;},\ldots ,\;y_{N\;}\right]}^{T}$ and N independent variables denoted as $X={\left[x_{1\;},\;x_{2\;},\ldots ,\;x_{N\;}\right]}^{T}$. Capital letters such as X were used to represent a random variable, and lower-case letters such as x to represent the value of the variable. Furthermore, vectors are denoted in bold letters. Regression aims to estimate the mathematical relation f() parameterized by a vector β to explain a dependent variable Y in terms of independent variables X.

(1) $$Y=f\left(X,\beta \right).$$

Regression aims to minimize a loss function l between the estimated values $f\left(x_{n},w\right)$ and the observed values $y_{n}$ for N training instances by estimating β.

(2) $$\beta =argmin\overset{N}{\underset{n=1}{\sum }}\;l\left(f\left(x_{n},\beta \right),\;y_{n}\right)$$

As the true function that maps X onto Y (plus noise) is unknown, different metrics, such as mean absolute error (MAE) are used to describe the performance of regression based on the residual of the predictions [32]. To select the regression model, the following machine learning algorithms for regression were tested in a preliminary study: SVM, linear regression, Gaussian kernel, Gaussian process regression (GPR), random forests, and decision trees. We chose GPR due to it having higher accuracy, stable results, and the possibility to specify the prediction interval.

### 2.5. Gaussian Process Regression (GPR)

For a univariate Gaussian random variable Z, the distribution is fully specified by the mean µ and standard deviation σ. For N independently distributed random variables $Z_{N}$ (k=1 , … , N), which can be organized into a random vector $Z={\left[Z_{1\;},\;Z_{2\;},\ldots ,\;Z_{N\;}\right]}^{T}$**,** the distribution is fully specified by the (N-dimensional) mean vector µ and (NxN-dimensional) covariance matrix Σ. The multivariate Gaussian distribution of the random vector Z is shown in the following equation.

(3) $$Z\;∼\;N\left(µ,\;\Sigma \right)$$

A Gaussian process (GP) generalizes a multivariate Gaussian distribution to infinitely many variables. This leads to a distribution, which is not distributed over functions instead of vectors. The mean function, the covariance function, and the GP of a random function $f\left(x\right)$ are shown in the following equation.

(4) $$µ=m\left(x\right)$$

(5) $$\Sigma =k\left(x,x^{\prime }\right)$$

(6) $$f\left(x\right)\;∼\;\mathrm{GP}\left(m\left(x\right),k\left(x,x^{\prime }\right)\right)$$

For simplicity, the mean vector can be assumed to be zero, although this is not always the case, as explained in the last section of this chapter. For simplification purposes, the relation of one observation to another is only defined by the covariance function. Decomposing a random function f arbitrarily into two subsets of function values a and b leads to the following equation where A, B, and *C* are the corresponding pieces that makeup Σ.

(7) $$f=\left[\begin{array}{c} a\\ b \end{array}\right]∼N(0,\;\left[\begin{array}{cc} A & C^{T}\\ C & B \end{array}\right])$$

When *C* is non-zero, a transformation of Equation (7) leads to the conditional distribution of *b* given *a*

(8) $$p\left(b|a\right)\;∼N\left(µ,\Sigma \right)$$

(9) $$µ=CA^{-1}a\;$$

(10) $$\Sigma =B-CA^{-1}C^{T}$$

In summary, if we know some of f (here a), we can use that to estimate the rest of f (here b). This is important to Gaussian process regression (GPR). In GPR, our goal is to predict the independent variable $Y^{*}$ of dependent variables $X^{*}$ in a new data set, based on a training data set with the independent variable $Y={\left[y_{1\;},\;y_{2\;},\ldots ,\;y_{N\;}\right]}^{T}$ ($y_{n\;}$: single value) and the dependent variables $X={\left[x_{1\;},\;x_{2\;},\ldots ,\;x_{N\;}\right]}^{T}$ ($x_{n\;}$: d-dimensional input vector) containing N training samples. The key idea of GPR is to model the response as a sample from a multivariate Gaussian distribution. More specifically, GPR models the response as a noise version of Gaussian distributed function values as shown in the following equation.

(11) $$Y=f\left(X\right)\;+\;\varepsilon$$

The distribution of function values is Gaussian, with the mean function of zero and covariance function *k* as well as the noise with zero mean and variance σ ($\varepsilon ∼\;N\left(0,\sigma^{2}I\right)$). This leads to the following equation.

(12) $$Y∼\;N\left(0,k\left(X,X\right)\;+\;\sigma^{2}I\right)$$

Furthermore, the extension of Equation (7) to the GPR with noise leads to the following equation.

(13) $$\left[\begin{array}{c} Y\\ Y^{*} \end{array}\right]\;∼N(0,\left[\begin{array}{cc} k\left(X,X\right)\;+\;\sigma^{2}I & k\left(X,X_{*}\right)\\ k\left(X_{*},X\right) & k\left(X_{*},X_{*}\right) \end{array}\right])$$

By deriving the conditional distribution $Y^{*}$ given Y corresponding to Equations (9)–(11), we can formulate the predictive equations for GPR. Our best estimate for $Y^{*}$ is the mean of this distribution, and the uncertainty in our estimate is captured in its variance.

(14) $$Y_{mean}^{*}=K\left(X_{*},X\right){\left[K\left(X,X\right)\;+\;\sigma^{2}I\right]}^{-1}Y$$

(15) $$cov\left(Y^{*}\right)\;=K\left(X_{*},X_{*}\right)-K\left(X_{*},X\right){\left[K\left(X,X\right)\;+\;\sigma^{2}I\right]}^{-1}-K\left(X,X_{*}\right)$$

The GPR model that was used was set up in MATLAB R2020b (The MathWorks, United States). To calculate the covariance function, ten different kernels were explored. A popular kernel, the “squared exponential”, is shown in Equation (16).

(16) $$k\left(x,x^{\prime }\right)\;=\sigma_{f}^{2}e^{\frac{-{\left(x-x^{\prime }\right)}^{2}}{2l^{2}}}$$

For this kernel, $\sigma_{f}^{2}$ is the maximum allowable covariance. If $x\approx x^{\prime }$, then the covariance function approaches this maximum. This means that $f\left(x\right)$ is highly correlated with $f\left(x^{\prime }\right)$. Besides the “squared exponential” kernel, one “exponential”, two “matern”, one “rational quadratic”, and five “ard” kernels were evaluated by parameter optimization. In this work, other than zero mean functions, non-zero mean functions were also used. The use of explicit basis functions is a way to specify a non-zero mean over functions. For parameter optimization, different basis functions (“constant”, “linear”, and “quadratic”) were investigated and used, which are described in [33].

### 2.6. Machine Learning Framework

**Figure 5:**
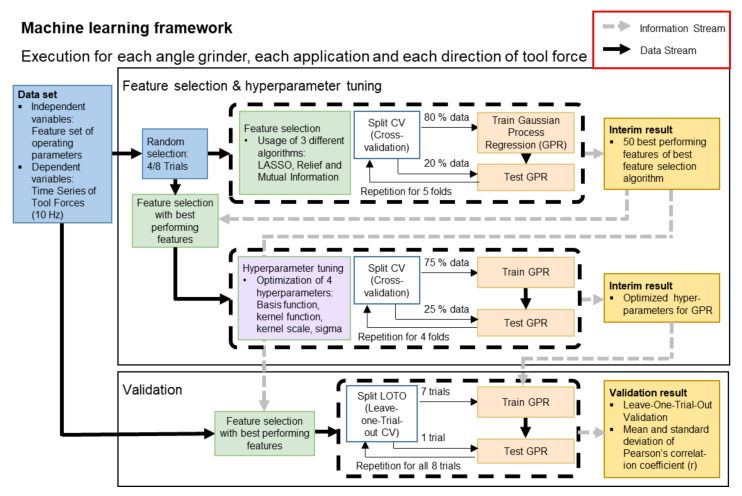
The machine learning framework used in this study. The framework is divided into a model and feature selection stage, in which the best model, features, and hyperparameters are identified, and a validation process, in which the best performing model is validated.

The following section describes the framework for finding a generalized machine learning model. This includes selecting well-performing features and regression models, fitting the hyperparameters to the training dataset, and validating the generalization of the chosen model on our dataset. The framework is shown in Figure 5.

The following framework is applied to both angle grinders, all four applications, and all force directions ($F_{guiding,fiber}$, $F_{tangential,fiber}$, $F_{normal,fiber}$, $F_{guiding,roughing}$,$\;F_{tangential,\;roughing}$, $F_{normal,roughing},\;F_{guiding,flap},F_{tangential,flap}$, $F_{normal,flap}$, $F_{axial,cut}$, and $F_{radial,cut}$). This aims at tailoring the features and regression model, including the hyperparameters, to the forces separately. The model selection procedure is based on a nested randomly chosen feature subset, which consists of 50% of the feature data. This approach is called nested CV. It allows to nest the selection and hyperparameter optimization procedure under the testing of selected features in the validation process. 

A reduction of the number of features helps to reduce the problem of high dimensionality and to maximize the accuracy of regression models. Therefore, the feature selection methods “Mutual Information”, “LASSO”, and “Relief” were used to rank all features by their importance and further select the 50 best features. The performance was assessed by 5-fold CV. To improve the performance of the GPR method, the hyperparameters were further tailored to the nested feature set. The following hyperparameters were optimized for GPR: Basis function (mean function), kernel function (covariance function), kernel scale, and sigma (noise). The optimization was carried out using a quasi-Newton procedure in 4-fold CV.

Validation of the regression model was performed using a specific type of leave-one-out CV, known as the leave-one-trial-out (LOTO) validation. To achieve this, the feature set was repeatedly split into a test set (one trial) and a training set (remaining trials). The segments of the training sets were further randomly ordered. Moreover, a regression model with the information from the generalization procedure (GPR with the best hyperparameters and the best 50 features) was trained on the training set. Assessment of the individually trained models for each of the trials was based on the error between predicted force time series ($F^{*}$) and measured-value force time series (F). To compare the prediction accuracy with the state of the art, the last validation step was also performed with 5-fold CV instead of a LOTO validation. Here, the test set was not formed from a single trial that might differ from the other trials, but from 20% randomly drawn segments of the data set containing all trials.

Since the absolute force value is of great importance to dimensioning, mean absolute error (MAE) was used as an evaluation parameter. To consider the relative deviation of the measured and predicted force values, the relative MAE (rMAE) was used as a second evaluation parameter. It is calculated by dividing the MAE value by the average of each tool force. In addition, the Pearson correlation coefficient (r) was used as a dimensionless evaluation parameter, which is a measure of linear correlation between the measured and predicted force values. The overall prediction accuracy for all trials and one tool force was calculated by the mean overall trials for each application and direction. Additionally, the standard deviation was calculated to quantify the variance among all trials.

## 3. Results

**Figure 6:**
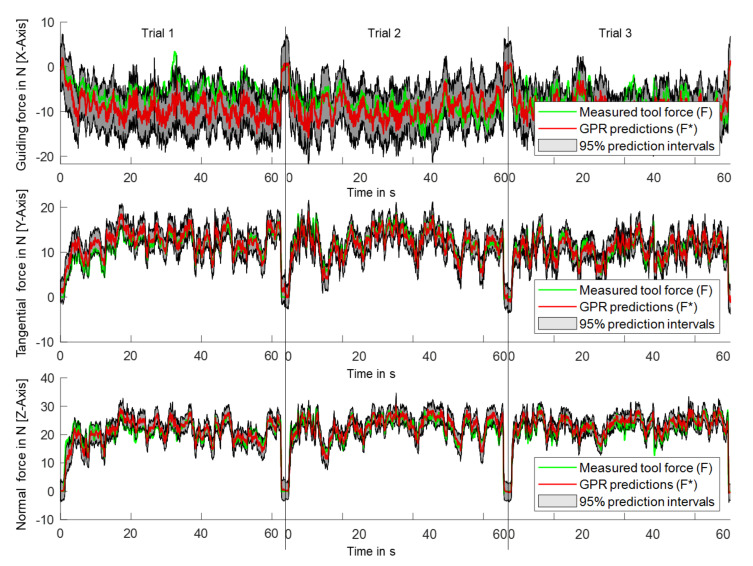
Time series of measured tool force and with GPR predicted tool forces including their 95% prediction intervals in all three directions for *roughing with a fiber disc* for three trials.

The time series of predicted ($F^{*}$) and measured (F) tool forces for the application roughing with a roughing disc in three directions ($F_{guiding,roughing}$, $F_{tangential,roughing}$, $F_{normal,roughing}$) in three trials are shown in Figure 6. Predictions for each of the trials were obtained from the leave-one-trial-out (LOTO) validation and plotted one after another. It can be seen that there is good agreement including peaks between the predicted and measured force curves for $F_{tangential,roughing}$ and $F_{normal,roughing}$. In addition, the figure shows the 5% and 95% prediction interval. The predicted time series for $F_{tangential,roughing}$, and $F_{normal,roughing}$ are consequently within the 95% prediction interval. The prediction interval for $F_{guiding,roughing}$ is higher than for $F_{tangential,roughing}$ and $F_{normal,roughing}$, which means that the probability of an accurate prediction for $F_{guiding,roughing}$ is lower.

The statistical analysis over all trials (N = 31) for the angle grinder GWS-18 is shown in Table 1. The predicted forces yielded MAE values in the range of 1.03 N to 6.84 N, rMAE values in the range of 9.42% to 630.23%, and r values in the range of 0.09 to 0.91. Furthermore, the prediction accuracy is classified into three groups (<15%, 15–30%, >30%) using the evaluation variable rMAE. The tool forces that were found to have an rMAE below 15% were $F_{normal.roughing}$, $F_{tangential.fiber}$, and $F_{normal.fiber}$. The tool forces with an rMAE between 15% and 30% were $F_{tangential,roughing}$, $F_{tangential.flap}$, $F_{normal.flap}$, and $F_{radial,cut}$. Lastly, the tool forces with an rMAE above 30% were $F_{guiding,roughing}$, $F_{guiding,fiber}$, $F_{guiding,flap},$ and $F_{axial,cut}$. 

The statistical analysis over all trials for the angle grinder CCG-18 (N = 32) is shown in Table 2. The predicted forces yielded MAE values in the range of 1.14 N to 7.87 N, rMAE values in the range of 7.52% to 503.67%, and r values in the range of 0.16 to 0.94. The prediction accuracy is classified into three groups (<15%, 15–30%, >30%) using the evaluation variable rMAE. The tool forces with an rMAE below 15% were $F_{normal.roughing}$, $F_{tangential.fiber}$, $F_{normal.fiber},\;F_{tangential.flap}$, and $F_{z.flap}$. The tool forces with an rMAE between 15% and 30% were $F_{tangential,\;roughing}$, and $F_{radial,cut}$. The tool forces with an rMAE above 30% were $F_{guiding,roughing}$, $F_{guiding,\;fiber}$, $F_{guiding,\;\;flap}$, and $F_{axial,\;cut}$.

The statistical analysis regarding the different accuracies due to the influence of the sampling frequency of the predicted tool force and the validation strategy is shown in Table 3 for the angle grinder GWS18. In the second column, the rMAE values of the previous tables with LOTO validation and sampling frequency of 10 Hz are shown. In the first and third columns, the tool forces are predicted with a sampling frequency of 1 Hz and 20 Hz to investigate the accuracy of higher and lower sampled tool forces. In the fourth column, the tool force is predicted at a sampling frequency of 10 Hz and the accuracy is validated using 5-fold CV instead of a LOTO validation. This was done to investigate the influence of the validation. In the fifth column, the accuracy is validated using 5-fold CV at a sampling frequency of 10 Hz with the evaluation parameter coefficient of determination (R^2^). This was done for a better comparison of the results with the other study [29] and the preliminary study of the authors [18].

Table 4 shows the statistical analysis regarding the different accuracies due to the influence of the sampling frequency of the predicted tool force and the validation strategy for the angle grinder CCG18 in the same way as Table 3.

## 4. Discussion

### 4.1. Discussion of the Results and Comparison of the Applications and Hand-Held Grinding Machines 

The results show a good accuracy for the radial force for *cutting with a cut-off wheel* (rMAE = 19.67% and r = 0.80) as well as for the grinding tangential force (rMAE = 18.21% and r = 0.82) and grinding normal force (rMAE = 11.44% and r = 0.84) for the other three applications. The high accuracies can be explained by the fact that the grinding normal force has a physical relation with the torque of the hand-held grinding machine and, thus, with the current of the motor of the grinding machine [19]. The grinding tangential force is dependent on the grinding normal force via the friction coefficient between tool and workpiece [1]. The prediction of the axial force for *cutting with a cut-off wheel* (rMAE = 566.95% and r = 0.13) seems not feasible. The accuracy of the prediction of the guiding force (rMAE = 87.02% and r = 0.37) is worse for the other three applications, which can be explained by the strong influence of the user on the guiding force by his motion and way of working. In addition, the force values in axial force direction are very low, so that the stochastic behavior of the abrasive process and the local oscillations of the tool have a greater influence than in the other force directions.

When comparing the applications, it is observed that the applications *roughing with a roughing disc* and *roughing with a fiber disc* have the best accuracy. This can be explained by the fact that the user essentially works in one direction, which can be seen in Figure 3. Due to the linear movement of the user, the pressure angle and the lateral movement only play a minor role, which supports the prediction based on the operating parameters. For the application *grinding with a flap disc*, the poorer results for the guiding and grinding tangential force can be explained by the multidimensional and more random motion in this application, hence leading to a poor assignment of the guiding force and the grinding tangential force to the force directions due to the fixed coordinate system of the test setup. Thus, the tool force to be predicted for the application *grinding with flap disc* is already biased. The better accuracy of the applications *roughing with a roughing disc* and *roughing with a fiber disc* can be explained by the much higher stiffness and lower damping of the roughing disc and fiber disc, thus, leading to a lower information loss between the tool-workpiece contact and the data logger. The application *cutting with a cut-off wheel*, however, differs much from the other ones. The pressure angle and contact are much more volatile. For *cutting with a cut-off wheel*, the axial forces in X-direction display the transverse oscillations, resulting in dynamic effects that cannot be predicted in this study with the data measured by the data logger. Since the grinding tangential force and grinding normal force could be predicted well in all tested applications, it can be assumed that the prediction of tool forces for other applications will also be successful, given that the regression model is specifically trained for this new application.

Increasing the segment length from 0.1 s to 1 s, and, thus, predicting a force sampled at a frequency of 1 Hz, results in a partially worse accuracy. This can be explained by the fact that the force components of the tool force, which occur due to the user’s motion, have a frequency of up to 10 Hz [34]. Therefore, these are included in the independent variable (operating parameters of the angle grinder) but not in the dependent variable (tool force) due to the filtering to 1 Hz. In addition, the number of segments is ten times smaller, which makes training GPR models more difficult. The prediction of tool forces with a sampling frequency of 20 Hz leads to a similar to slightly worse accuracy. This can be explained by the fact that the prediction of dynamic tool forces and their effects is more difficult than the prediction of less dynamic forces, especially when only time series with a frequency of 1000 Hz are available as independent variables. For the prediction of more dynamic tool forces, the importance of features in the frequency domain is particularly high. The shorter segment length means that the amount of data in a segment is smaller, which results in a lower frequency resolution of the FFT. This means that many features in the frequency domain cannot be used meaningfully at smaller segment lengths for low-frequency operating signals of 1000 Hz. An improvement would be a higher frequency recording of the operating signals in order to obtain better features in the frequency domain also for small segment lengths.

When comparing the accuracy of both angle grinders, it can be seen that both have a roughly similar accuracy, although they have considerably different absolute values for the tool forces and also considerably different absolute values for the measured variables current, acceleration, and magnetic field. This suggests that the approach and models are transferable to other angle grinders. The transferability to other hand-held grinders such as a rotary grinder should be further investigated. 

The performance of the GPR was tested with a variety of kernels, mean functions, and hyperparameters in the machine learning framework in a multi-stage process. Due to the multi-stage process, it can be assumed that the accuracy of prediction using GPR was largely exploited in this data set. In comparison with SVM, linear regression, Gaussian kernel, random forests, and decision trees, GPR showed superiority in a preliminary study in terms of accuracy and stability of results. Due to the variety of algorithms available for regression, it cannot be excluded that other methods such as neural networks, which have been successfully used for similar problems [23,24,35,36,37], would have led to even better accuracy. However, especially for deep learning methods with a very large number of parameters, a large data set must be available to demonstrate the transferability of the results.

### 4.2. Comparison of the Result with the State of the Research

The comparison of the results with the preliminary study by the authors [18] shows a decrease in accuracy for the tangential force in the Y-direction (R^2^ = 0.91 vs. R^2^ =0.79) and normal force in the Z-direction (R^2^ = 0.97 vs. R^2^ =0.78) compared to this study. The low accuracy for the guiding force and axial force in the X-direction compared to the other force directions is consistent with this study (R^2^ = 0.33, R^2^ =0.42). This corresponds to the expected result since the change from wired laboratory measurement equipment to the consumer-grade sensors in the data logger for real-world usage leads to a degradation of the signal quality. This can be explained by the lower sampling frequency (1000 Hz vs. 20,000 Hz) and the lower resolution and signal-to-noise ratio of the IMU, current, and voltage sensor of the consumer-grade sensors. Moreover, a great influence on the signal quality is the location and attachment of the sensor in relation to the origin of the tool forces, which is the tool-workpiece contact. While in the preliminary study [18], the accelerometer was screwed rigidly to the gearbox housing, the IMU located at the battery was much further away from the tool-workpiece contact and, therefore, much less rigidly connected due to the polymer housing of the data logger in this study. For manual grinding, single tests are not identical, as also shown by the standard deviation of the prediction and Figure 6. Therefore, the use of LOTO validation in this study leads to a significant decrease in accuracy but a significant increase in the transferability of the prediction model compared to the CV of the preliminary study. This can also be seen by comparing the results from the LOTO validation with the CV from Table 3 and Table 4. Here, especially in the X-direction, there are significantly lower rMAE values for the CV, which correspond to the results from the preliminary study [18].

The comparison with the prediction model based on a binary classification of force ranges [29] shows a similar accuracy grade. Again, the use of LOTO validation in this study results in a higher transferability in contrast to the CV. As an improvement, [29] proposes the division into force ranges with lower resolution. Since the force ranges are physically related and the regression approach allows the division into force ranges in post-processing, the authors consider the regression approach superior for the prediction of the tool force.

The comparison with the prediction of tool forces using machine learning in automated grinding shows higher accuracy with automated grinding in most cases. This is in line with the expected result that the forces in the manual grinding process, which are hand-held, are much more volatile since they are influenced by the user’s motion, gripping force, and user forces [12]. Hence, they are more difficult to predict. Other influences such as the wear of the grinding disc [1], state of charge of the battery in hand-held cordless grinders, and lower stiffness of the tools complicate the prediction. The comparison with the prediction of body forces and movements such as knee joint forces (KJF) with one or more IMUs shows a similar approach (leave-one-out cross-validation and classical and deep learning approaches) for regression [23,24,25,26,27,28]. Similar accuracies are obtained, for example r = 0.60 to r = 0.94 for vertical KJF, r = 0.64 to r = 0.90 for anterior-posterior KJF, r = 0.25 to r = 0.60 medial-lateral KJF [23]) despite a different system being investigated, and more scatter is caused by the grinding machine.

### 4.3. Limitations and Further Research Directions

The results indicate a strong dependency of the prediction model on the chosen factors angle grinders, tool types, and force directions, since partially different features and hyperparameters were obtained as interim results for both angle grinders, the four tool types, and the three force directions. This indicates that different prediction models must be trained and employed for different angle grinders, different applications, and different force directions. The findings correspond to the expected results since the overall system is determined by the hand-held machines (e.g., by the drive train, the gearing, the bearing, the control algorithms) and different tool types (e.g., by the stiffness and damping coefficient due to the structure and material). Furthermore, the influence of different tool types on the dynamic part of the tool forces or the dynamic power transmission has been shown in [38]. To employ the prediction model, it will be necessary to parameterize the model specifically for a hand-held machine in combination with the tool type for each force direction. A challenge for real-world application will be to identify the tool in order to select the appropriate model for the prediction of the tool forces. This requires either information from the user who uses the tool or from classification using machine learning. First approaches to the classification of the tool type or application have been published already [15,29,39,40,41]. 

The further transferability of the results is always dependent on a diverse and representative data set. Therefore, appropriate factors regarding the application (four tool types) and hand-held grinding machine (two angle grinders) were chosen to estimate the transferability. The study was deliberately executed by one test person since the correlation between the tool forces from the internal operating variables is determined by the operating point of the machine. The operating point is indirectly influenced by a user via pressure angle, movement, and user forces. Thus, the question of whether the trials in the data set contain enough operating points for transferability to other users arises. A good transferability regarding the user can be assumed, since the usual operating range of the angle grinder (speed and operating forces) was exhausted by the number and especially the length of the trials in the manual tests. Figure 6 shows the variability of the forces and, thus, the exhaustion of the usual operating range. One workpiece type per tool type or application was used. Therefore, further studies need to examine how transferable the prediction with the same tool is to other workpieces. This poses a particular challenge since the verification of the prediction is only possible by comparing the measured tool force with the predicted force values. The tests were carried out in a proper manner by an experienced user. Another research direction is to extend the prediction to misuse cases where the working process deviates significantly from working in a proper manner (e.g., fluttering of the tool due to very low-pressure force). 

Since there are already promising results in predicting tool forces with machine learning in automated grinding, there is still great potential and further research directions [8]. Future work for the prediction of tool forces includes the reduction of uncertainty and new applications as new work materials [7]. This is even more applicable to the prediction of tool forces in manual grinding since the prediction in this application is more challenging, and only a few studies investigate this method [14]. An important further research direction besides the development of prediction models is the development and integration of sensors in hand-held grinding machines to gather more process data since this offers a possibility to eliminate the disadvantage of the large distance of the sensors of the data logger to the tool-workpiece contact.

The model was used to predict the tool forces sampled with 10 Hz, 1 Hz, and 20 Hz. A further research direction is the prediction of the tool forces sampled at a higher frequency. With a higher sampling frequency of operating parameters and more sensors, such as a microphone, the information value could be increased, so that a prediction of the tool forces with a higher sampling may be feasible. An alternative to the prediction of the time signals of the forces with higher sampling frequencies is the prediction of certain amplitude values in the frequency spectrum of the tool force. This would be well suited, for example, for the amplitude values of the rotational speed orders of both shafts, since these account for a large part of the dynamic tool force.

## 5. Conclusions

In this study, the feasibility of predicting the tool forces at 10 Hz from the measured data of consumer-grade sensors such as an IMU, a current sensor, and a voltage sensor on a hand-held angle grinder was investigated. The results indicate a good prediction of the grinding normal force (rMAE = 11.44% and r = 0.84) and grinding tangential force (rMAE = 18.21% and r = 0.82) for three tested applications as well as for the radial force for *cutting with a cut-off wheel* (rMAE = 19.67% and r = 0.80). The prediction of the guiding force (rMAE = 87.02% and r = 0.37) perpendicular to the grinding tangential force and grinding normal force and the axial force for *cutting with a cut-off wheel* (rMAE = 566.95% and r = 0.13) seems not feasible. Hence, it is evident that these forces, which are mainly determined by the user, cannot yet be predicted well. Transferability of the accuracy of the tool force prediction and the approach to other angle grinders was demonstrated using two different angle grinders. For the application of the approach, it must be considered that a specific regression model needs to be trained for each hand-held grinding machine, each application, and each force direction.

The prediction of tool forces with the proposed GPR model enables the potential of data acquisition and evaluation using consumer-grade sensors in real-world applications. The achieved accuracy in combination with the prediction interval using the proposed GPR model enables the use of the tool force data for the development and dimensioning of hand-held grinding machines since the probability of the prediction interval can be integrated into the dimensioning. In addition, the accuracy of the tool force prediction enables the development and deployment of optimized control algorithms and predictive models, such as a predictive maintenance model or an estimation model of material removal and machined surface quality, in combination with the proposed model.

## Figures and Tables

**Table 1:** Prediction of the tool forces for the angle grinder GWS-18 for a sampling frequency of 10 Hz. The accuracy is presented as mean (and standard deviation) with the mean-absolute-error (MAE), the relative mean-absolute-error (rMAE), and the Pearson’s correlation coefficient (r).

Application	MAE [N]	rMAE [%]	r	MAE [N]	rMAE [%]	r	MAE [N]	rMAE [%]	r
Roughing with a roughing disc	$F_{guiding,roughing}$			$F_{tangential.roughing}$			$F_{normal.roughing}$		
	2.83 (0.64)	137.25 (67.77)	0.47 (0.12)	1.66 (0.43)	20.91 (4.09)	0.85 (0.04)	2.15 (0.56)	9.42 (2.31)	0.91 (0.04)
Roughing with a fiber disc	$F_{guiding,fiber}$			$F_{tangential.fiber}$			$F_{normal.fiber}$		
	2.64 (0.52)	64.38 (42.52)	0.48 (0.11)	1.03 (0.21)	11.11 (2.43)	0.89 (0.05)	1.80 (0.36)	10.06 (1.57)	0.80 (0.19)
Grinding with a flap disc	$F_{guiding,flap}$			$F_{tangential.flap}$			$F_{normal.flap}$		
	6.84 (1.77)	108.93 (67.21)	0.10 (0.19)	2.28 (0.73)	27.71 (16.37)	0.59 (0.14)	3.79 (1.67)	25.34 (16.83)	0.58 (0.16)
Cutting with a cutoff-wheel	$F_{axial.cut}$			$F_{radial.cut}$					
	3.14 (1.08)	630.23 (888.75)	0.09 (0.14)	4.66 (2.99)	24.03 (9.88)	0.70 (0.05)			

**Table 2:** Prediction of the tool forces for the angle grinder CCG18 for a sampling frequency of 10 Hz. The accuracy is presented as mean (and standard deviation) with the mean-absolute-error (MAE), the relative mean-absolute-error (rMAE), and the Pearson’s correlation coefficient (r).

Application	MAE [N]	rMAE [%]	r	MAE [N]	rMAE [%]	r	MAE [N]	rMAE [%]	r
Roughing with a roughing disc	$F_{guiding,roughing}$			$F_{tangential.roughing}$			$F_{normal.roughing}$		
	2.63 (0.78)	84.47 (16.62)	0.17 (0.17)	1.69 (0.40)	23.27 (5.47)	0.81 (0.06)	2.01 (0.38)	8.68 (1.67)	0.85 (0.21)
Roughing with a fiber disc	$F_{guiding,fiber}$			$F_{tangential.fiber}$			$F_{normal.fiber}$		
	2.94 (0.90)	39.04 (7.08)	0.58 (0.07)	1.14 (0.25)	12.32 (4.67)	0.91 (0.07)	1.66 (0.27)	7.62 (1.01)	0.94 (0.02)
Grinding with a flap disc	$F_{guiding,flap}$			$F_{tangential.flap}$			$F_{normal.flap}$		
	7.87 (0.99)	88.06 (54.40)	0.41 (0.11)	1.67 (0.37)	13.97 (4.40)	0.83 (0.08)	1.81 (0.40)	7.52 (1.71)	0.94 (0.02)
Cutting with a cutoff-wheel	$F_{axial.cut}$			$F_{radial.cut}$					
	4.62 (2.04)	503.67 (532.58)	0.16 (0.16)	2.61 (0.42)	15.31 (3.63)	0.90 (0.04)			

**Table 3:** Prediction of the tool forces for the angle grinder GWS18 for different sampling frequencies. The accuracy of the prediction is presented as mean (and standard deviation) with the relative mean-absolute-error (rMAE) for a sampling frequency of 1 Hz, 10 Hz, 20 Hz, and for CV instead of a LOTO-validation for a sampling frequency of 10 Hz. The coefficient of determination (R^2^) is also shown for the CV for a sampling frequency of 10 Hz.

Application	rMAE [%]				R^2^	rMAE [%]				R^2^	rMAE [%]				R^2^
	1 Hz	10 Hz	20 Hz	CV	CV	1 Hz	10 Hz	20 Hz	CV	CV	1 Hz	10 Hz	20 Hz	CV	CV
Roughing with a roughing disc	$F_{guiding,roughing}$					$F_{tangential.roughing}$					$F_{normal.roughing}$				
	131.60 (75.57)	137.25 (67.77)	136.87 (69.77)	62.90	0.58	23.73 (3.10)	20.91 (4.09)	20.82 (3.58)	15.38	0.81	12.79 (4.95)	9.42 (2.31)	9.41 (2.73)	7.63	0.87
Roughing with a fiber disc	$F_{guiding,fiber}$					$F_{tangential.fiber}$					$F_{normal.fiber}$				
	71.57 (51.55)	64.38 (42.52)	62.99 (40.15)	50.66	0.26	14.27 (3.54)	11.11 (2.43)	12.62 (2.88)	9.14	0.86	11.42 (1.03)	10.06 (1.57)	10.90 (2.41)	11.85	0.69
Grinding with a flap disc	$F_{guiding,flap}$					$F_{tangential.flap}$					$F_{normal.flap}$				
	100.82 (69.40)	108.93 (67.21)	102.40 (64.12)	75.31	0.24	80.61 (109.90)	27.71 (16.37)	71.59 (106.04)	13.92	0.68	30.49 (24.28)	25.34 (16.83)	31.96 (12.95)	10.91	0.71
Cutting with a cutoff-wheel	$F_{axial.cut}$					$F_{radial.cut}$									
	490.22 (517.88)	630.23 (888.75)	644.98 (886.18)	624.30	0.24	19.94 (11.69)	24.03 (9.88)	23.34 (9.00)	14.87	0.76					

**Table 4:** Prediction of the tool forces for the angle grinder CCG18 for different sampling frequencies. The accuracy of the prediction is presented as mean (and standard deviation) with the relative mean-absolute-error (rMAE) for a sampling frequency of 1 Hz, 10 Hz, 20 Hz, and for CV instead of a LOTO-validation for a sampling frequency of 10 Hz. The coefficient of determination (R^2^) is also shown for the CV for a sampling frequency of 10 Hz.

Application	rMAE [%]				R^2^	rMAE [%]				R^2^	rMAE [%]				R^2^
	1 Hz	10 Hz	20 Hz	CV	CV	1 Hz	10 Hz	20 Hz	CV	CV	1 Hz	10 Hz	20 Hz	CV	CV
Roughing with a roughing disc	$F_{guiding,roughing}$					$F_{tangential.roughing}$					$F_{normal.roughing}$				
	78.12 (32.19)	84.47 (16.62)	82.18 (20.77)	52.03	0.71	24.73 (2.95)	23.27 (5.47)	23.09 (6.48)	15.33	0.81	12.02 (1.49)	8.68 (1.67)	8.59 (1.11)	7.67	0.87
Roughing with a fiber disc	$F_{guiding,fiber}$					$F_{tangential.fiber}$					$F_{normal.fiber}$				
	38.99 (6.89)	39.04 (7.08)	40.05 (11.08)	37.53	0.58	23.92 (11.36)	12.32 (4.67)	14.30 (5.94)	7.97	0.89	12.03 (3.28)	7.62 (1.01)	8.37 (1.14)	7.07	0.87
Grinding with a flap disc	$F_{guiding,flap}$					$F_{tangential.flap}$					$F_{normal.flap}$				
	83.18 (52.71)	88.06 (54.40)	95.59 (58.97)	65.92	0.41	16.65 (4.80)	13.97 (4.40)	13.95 (4.12)	13.49	0.71	9.23 (2.32)	7.52 (1.71)	8.10 (1.62)	10.83	0.71
Cutting with a cutoff-wheel	$F_{axial.cut}$					$F_{radial.cut}$									
	715.05 (1161.6)	503.67 (532.58)	529.96 (557.62)	553.79	0.36	19.83 (5.49)	15.31 (3.63)	19.26 (5.64)	14.86	0.76					

## Data Availability

The data that support the findings of this study are available from the corresponding author upon reasonable request.

## Abbreviations

The following abbreviations are used in this manuscript:

CV	cross validation
GP	Gaussian process
GPR	Gaussian process regression
KJF	knee joint forces
IMU	inertial measurement unit
LOTO	leave-one-trial-out
MAE	mean absolute error
r	Pearson correlation coefficient
R^2^	coefficient of determination
rMAE	relative mean absolute error
SVM	support vector machine
